# A study of hepatic hydatid cysts, focusing on cystobiliary fistula formation in children

**DOI:** 10.1007/s00383-025-06041-7

**Published:** 2025-05-16

**Authors:** Kamuran Tutuş, Selcan Türker Çolak, Şeref Selçuk Kılıç, Önder Özden, Derya Alabaz, Mehmet Emin İnal, Recep Tuncer

**Affiliations:** 1 Department of Pediatric Surgery, Faculty of Medicine, Çukurova University, Sarıçam, 01330 Adana, Turkey; 2 Department of Pediatric Infection, Faculty of Medicine, Çukurova University, Adana, Turkey; 3 Department of Radiology, Faculty of Medicine, Çukurova University, Adana, Turkey

**Keywords:** Hepatic hydatid cyst, Cystobiliary fistula, Pediatric hydatid disease, Biliary complications

## Abstract

**Purpose:**

Hydatid cysts, caused by *Echinococcus* species, represent a significant global health issue, particularly in endemic regions. Hepatic hydatid cysts may result in cystobiliary fistulas in 2–42% of cases, complicating treatment and leading to prolonged hospital stays and increased costs. This study aims to identify risk factors for cystobiliary fistula formation and evaluate outcomes in pediatric patients.

**Methods:**

We conducted a retrospective study involving 53 pediatric patients treated for hepatic hydatid cysts between 2002 and 2016. Patients were divided into two groups: the Fistula-Positive (FP Group, *n* = 15) and the Fistula-Negative (FN Group, *n* = 38). Cysts were classified as Fistulous Cysts (FC Group, *n* = 23) or Non-Fistulous Cysts (NFC Group, *n* = 92). We analyzed data regarding demographics, cyst characteristics, management, and outcomes.

**Results:**

Larger cyst size was significantly linked to the formation of cystobiliary fistulas (*p* = 0.040). Fistulous cysts had a median diameter of 100 mm, compared to 80 mm for non-fistulous cysts. Other factors, such as patient age, gender, residence, and cyst type, did not show a significant association. Patients with fistulas had a more extended hospital stay (median: 32 days vs. 7 days, *p* < 0.0001).

**Conclusions:**

A larger cyst size significantly predicted cystobiliary fistulas in hepatic hydatid disease. Proactive management, including advanced imaging, intraoperative techniques, and multidisciplinary care, is crucial for improving patient outcomes and minimizing complications of hepatic hydatid cysts, including cystobiliary fistulas.

## Introduction

Hydatid cysts, caused by the larval stages of cestodes from the genus *Echinococcus*, represent one of the most significant zoonotic diseases globally [1, 2]. They affect over one million people annually, resulting in healthcare costs exceeding $3 billion worldwide [3]. The disease is endemic in regions such as the Mediterranean, the Middle East, Central Asia, South America, and Africa, where its incidence varies from 2 to 200 per 100,000 individuals [2].

Hydatid cysts can form in various organs, with the liver being the most commonly affected, accounting for 60–70% of cases [2, 4, 5]. Differential diagnosis of cystic lesions should include this disease, considering the liver, the lungs, and other relevant sites in endemic regions [6]. In 1981, Gharbi provided a comprehensive classification system for liver hydatid cysts based on ultrasonographic findings. This system categorizes cysts into five distinct types, designated as follows: Type 1, characterized by a pure fluid collection with variable wall thickness; Type 2, distinguished by a membrane separated from the cyst wall; Type 3, exhibiting a honeycomb appearance and/or vesicles; Type 4, featuring a heterogeneous solid mass; and Type 5, displaying a heterogeneous calcified lesion [7]. Subsequently, the World Health Organization-Informal Working Group on Echinococcosis (WHO IWGE) refined and standardized this classification, adding a cystic lesion (CL) stage to the Gharbi classification and redefining Type 2 and Type 3 interchangeably. In addition, Type 3 was further delineated as cystic echinococcus (CE) 3a and CE 3b (source: Gharbi and WHO IWGE) [2].

A critical complication associated with hepatic hydatid cysts is their relationship with the biliary system, which can lead to cystobiliary fistulas in 2–42% of instances. If a cystobiliary fistula persists after treatment for hydatid cysts, it can result in prolonged hospitalizations and increased treatment costs due to the need for interventions such as endoscopic retrograde cholangiopancreatography (ERCP), biliary stenting, or surgical repair [8–10].

Understanding the factors contributing to cystobiliary fistula formation is essential for effectively managing hepatic hydatid cysts, especially in pediatric patients [11]. This study sought to determine risk factors for cystobiliary fistula formation in patients with hepatic hydatid cysts and to assess clinical outcomes and treatment approaches. 

## Materials and methods

### Study design

This retrospective study analyzed the records of pediatric patients (< 18 years) treated for hepatic hydatid cysts at our institution between June 2002 and June 2016. The study was based on the thesis *“Surgical, Percutaneous Radiological Intervention, and Benzimidazole Therapy in Childhood Hydatid Cysts* [12].*”*

Patients treated exclusively with albendazole or managed conservatively were excluded, as their cystobiliary fistula status could not be determined. The final cohort consisted of 53 patients undergoing surgical or percutaneous intervention. 

### Data collection

**Fig. 1:**
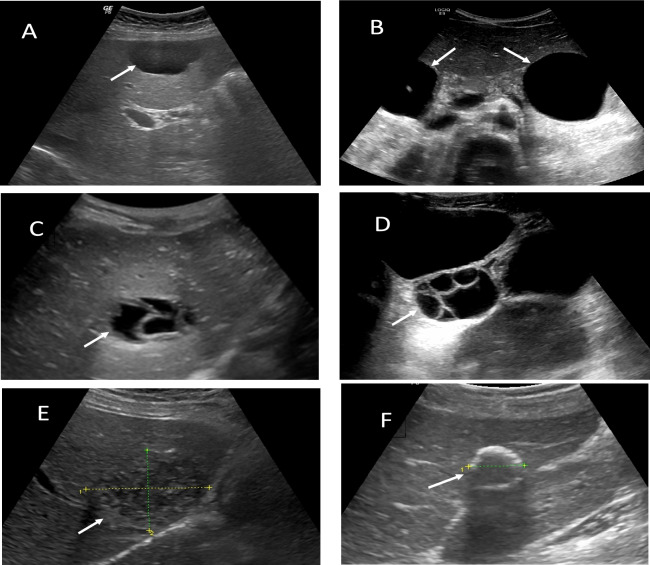
Ultrasonographic appearances of hepatic hydatid cysts according to Gharbi's classification (cysts are shown with arrows): **A & B**. Type 1; Pure fluid collection, **C**. Type 2; Fluid collection with a split wall, **D**. Type 3; Fluid collection with septa, honeycombed or with daughter vesicles **E**. Type 4; Reflecting thick walls, heterogeneous solid mass **F**. Type 5; Heterogeneous echo patterns, calcified hyperechoic lesion

Demographic, clinical, and procedural data were extracted from patient records. Cysts were classified according to the Ultrasonographic Gharbi classification system (Fig. 1), with the largest cyst dimension recorded [7]. The location of cysts in the liver was meticulously documented as either right, left, or bilateral. The respective segments in which these cysts were situated were also recorded, adhering to the established Couinaud classification system [13]. Serological test results [hydatid cyst indirect hemagglutination (IHA), Fumouze Diagnostics, France; positive if ≥ 1:320] were also documented.

#### Diagnosis of cystobiliary fistula

**Fig. 2:**
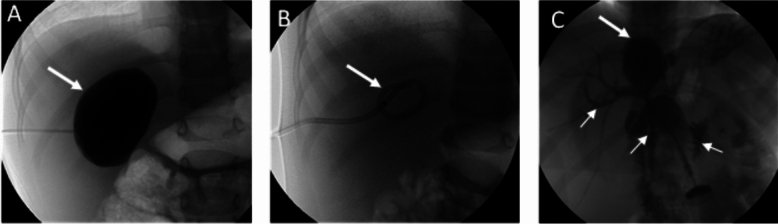
Examples of percutaneous interventions performed on Gharbi Type 1 cysts, categorized ultrasonographically. Images obtained under fluoroscopy during the percutaneous radiologic intervention: **A**. Cystogram (no cystobiliary fistula—the contrast material administered into the cyst marked with an arrow only filled the cyst without passing into the biliary system); **B**. Cystogram after the catheter was inserted into the cyst; **C**. Cystogram after the catheter was inserted into the cyst; C. Cystogram (the contrast medium introduced into the cyst marked with a thick arrow is seen to pass through the intrahepatic biliary ducts and common bile duct marked with thin arrows to the intestinal system (cystobiliary fistula present)

Cystobiliary fistulas were identified intraoperatively or during percutaneous interventions based on the following criteria:

o *Surgical cases*: Cystobiliary fistulas were identified according to the presence of bile in the fluid extracted from the cyst puncture during surgery, the presence of visible bile duct openings into the cyst cavity when the cyst was removed, postoperative bile drainage, or biloma formation. The diagnosis was confirmed through examination of surgical specimens from patients who underwent radical surgery.
o *Percutaneous cases*: PAIR (Puncture, Aspiration, Injection, Re-aspiration) or modified catheter techniques were used for percutaneous treatment of hydatid cysts [14]. Criteria for the presence of cystobiliary fistula were detection of bile during cyst puncture, visualization of the biliary tree on cystography, or subsequent bile drainage from the catheter (Fig. 2).

#### Patient and cyst grouping

Patients were categorized as:

o *Fistula-Positive Patients (FP Group)*: Patients with at least one cystobiliary fistula.
o *Fistula-Negative Patients (FN Group)*: Patients without any cystobiliary fistula.

Cysts were grouped as follows:

o * Fistulous Cysts (FC Group)*: Cysts with cystobiliary fistulas.
o *Non-Fistulous Cysts (NFC Group)*: Cysts without fistulas.

A comparative analysis was conducted on demographic data, serologic test results, the prevalence of hydatid cysts across different organs, the number of cysts in the liver, localization of the cyst in the liver, duration of post-procedural and total albendazole use, comorbidity data, the duration of hospitalizations (including both post-procedural stays and subsequent hospitalizations due to complications), and follow-up periods between the two patient groups. Furthermore, the study sought to ascertain whether there were any disparities in the type and size of cysts between the two cyst groups.

Pathological examination of the surgical and percutaneous treatment specimens confirmed the diagnosis of hydatid cysts. 

### Statistical analysis

Categorical variables were expressed as numbers and percentages, whereas continuous variables were summarized as mean, standard deviation, median, and minimum–maximum where appropriate. The chi-square test was used to compare categorical variables between the groups. The Shapiro–Wilk test confirmed the normality of distribution for continuous variables. Student’s t–test or Mann–Whitney U test was used to compare continuous variables between two groups depending on whether the statistical hypotheses were fulfilled. Logistic regression analysis was performed to determine significant predictors of cystobiliary fistula presence. In univariate analysis, variables significant at the *p* < 0.25 level and/or clinically significant variables were entered in multiple logistic regression analysis(Backward procedure). All analyses were performed using the IBM SPSS Statistics Version 20.0 statistical software package. The statistical level of significance for all tests was considered to be 0.05 [15].

## Results

### Patient demographics and clinical characteristics

During the study period, 71 patients were treated for hepatic hydatid cysts. Sixteen patients who received albendazole therapy alone and two who were followed without treatment were excluded from the study, leaving 53 patients whose biliary involvement could be evaluated. In addition to albendazole therapy, initial treatments included surgical intervention in 38 patients and percutaneous radiological procedures in 15 patients.

**Fig. 3:**
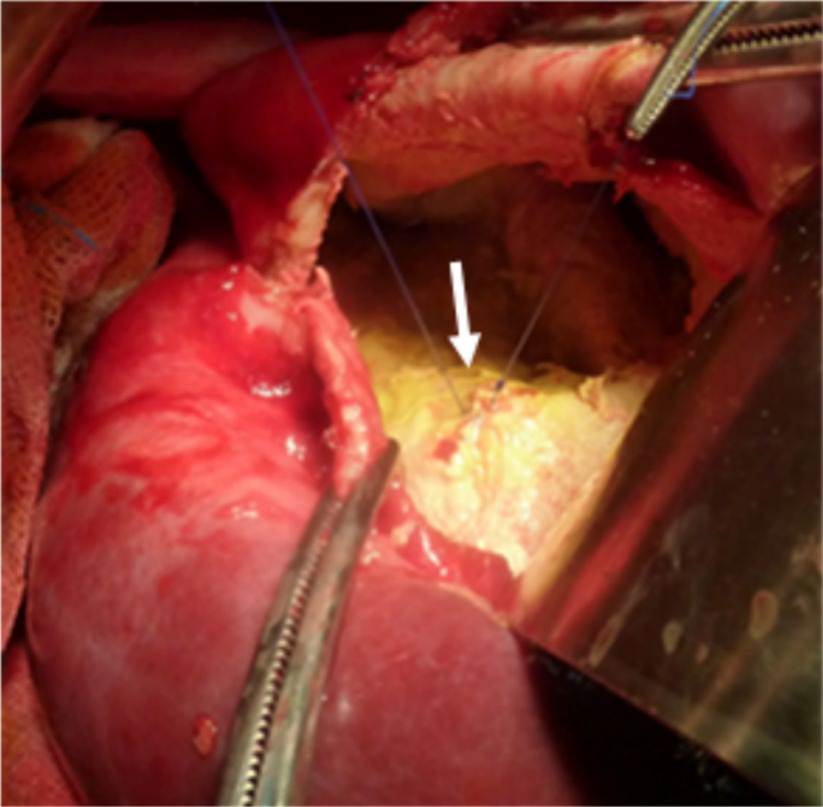
Operative view of a hepatic hydatid cyst with cystobiliary fistula. The arrow shows the repair of the bile duct that opened into the cyst cavity after endocystectomy

Among the 38 patients who underwent surgical treatment, 10 had intraoperative biliary fistulas repaired during the surgery (Fig. 3). No biliary fistula was identified during surgery in the remaining 28 patients; however, one of these patients developed bile drainage through the surgical drain postoperatively, leading to a diagnosis of biliary fistula. In the percutaneous radiological group, biliary fistulas were not detected in 11 patients, while biliary communication was observed in 4 patients. Two patients underwent radical surgery, and examination of the surgical specimens confirmed the presence of cystobiliary fistulas (one patient's cystobiliary fistula was radiologically identified pre-operatively, and the diagnosis was confirmed during specimen examination).

Biliary fistulas were detected in the liver cysts of 15 patients (FP Group), while no biliary fistulas were identified in the cysts of the remaining 38 patients (FN Group). The mean age of patients in the FP Group was 10.7 ± 4.0 years, compared to 9.8 ± 3.5 years in the FN Group. The FP Group included 6 females and 9 males, while the FN Group included 12 males and 26 females; there were no statistically significant differences in age and gender distribution (*p* = 0.434 and *p* = 0.794, respectively). Five patients in the FP Group and 12 in the FN Group resided in rural areas, with no significant difference between patient groups based on residence (*p* > 0.999). The median number of hydatid cyst-infected organs was 1 (1–2) in the FP Group and 1 (range: 1–4) in the FN Group. The median number of liver cysts was 2 (range: 1–4) in the FP Group and 1 (range: 1–13) in the FN Group. These differences were insignificant (*p* = 0.220 and *p* = 0.307, respectively). Cyst size was 102.2 ± 25.86 mm in FP patients and 83.45 ± 29.48 mm in FN patients (*p* = 0.040). In terms of the risk of cystobiliary fistula, 13 (86.7%) patients in the FP group and 22 (57.9%) patients in the FN group had cyst diameters above 75 mm (*p* = 0.95) (size in categorical evaluation: OR: 4.7, 95%CI: 0.9–23.9, *p* = 0.061). When the localization of cysts was analyzed regarding lobar distribution, 12 (80%) patients in the FP group had right-sided cysts, 1 (6. 7%) left, and 2 (13.3%) were bilateral; in the FN group, 26 (68.4%) patients had cysts in the right, 5 (13.2%) in the left, and 7 (18.4%) were located in bilateral lobes (*p* = 0.682). In the FP Group, there were 30 cysts, of which 23 demonstrated cystobiliary fistulas. In the FN Group, there were 85 cysts, all without biliary fistulas. Regarding comorbidities, two patients in the FP Group and eight patients in the FN Group had additional disorders, with no significant difference between the two groups (*p* = 0.706). Demographic and clinical characteristics of the patients according to fistula groups (FP and FN Groups), together with inter-group statistical comparison results, are presented in Table 1.

**Table 1:** Demographic and clinical characteristics of the patients according to fistula groups

	Patient Groups		*p-value*
	FP Group (*n* = 15)	FN Group (*n* = 38)	
Age (years)	10.7 ± 4.0	9.8 ± 3.5	0.434
Gender, n (%)			
Male	6 (40.0)	12 (31.6)	0.794
Female	9 (60.0)	26 (68.4)	
Place of residence n (%)			
Rural	5 (33.3)	12 (31.6)	> 0.999
Urban	10 (66.7)	26 (68.4)	
Number of organs with hydatid cysts	1.0 (1.0–2.0)	1.0 (1.0–4.0)	0.220
Number of liver cysts per patient at diagnosis	2.0(1.0–4.0)	1.0(1.0–13.0)	0.307
Cyst dimension (mm)	102.2 ± 25.86	83.45 ± 29.48	0.040
Cyst size, n(%)			
≤ 75	2(13.3)	16(42.1)	0.095
> 75	13(86.7)	22(57.9)	
Cyst location in the liver n(%)			
Right	12(80.0)	26(68.4)	0.682
Left	1(6.7)	5(13.2)	
Bilateral	2(13.3)	7(18.4)	
Total number n (FC/NFC)	30 (23/7)	85 (0/85)	
Albendazole duration (post-procedural)	232 (30–353)	120 (30–359)	0.116
Albendazole duration (total)	180 (90–360)	180 (30–360)	0.510
Comorbidity n(%)			
Absent	13(86.7)	30(78.9)	0.706
Present	2(13.3)	8(21.1)	
Symptom n(%)			
Absent	2(13.3)	7(18.4)	> 0.999
Present	13(86.7)	31(81.6)	
IHA, n(%)			
Not Tested	1(6.7)	4(10.5)	0.850
Negative	3(20.0)	9(23.7)	
Positive	11(73.3)	25(65.8)	
Hospital Stay (days)	32 (15–181)	7 (1–15)	< 0.001
Follow-up Duration (months)	25.0(4.0–118.0)	26.0(5.0–118.0)	0.465

Data were expressed as mean ± standard deviation or median(min–max) unless otherwise specified

*FP* Fistula-positive, *FN* Fistula-negative, *FP* Fistulous cyst, *NFC* Non-fistulous cyst, *IHA* Indirect hemagglutination test

### Cyst sizes, types, and locations

Across all patients, a total of 115 liver cysts, of which 23 were in the FC Group, and 92 were in the NFC Group. The median diameter of fistulous cysts was 100 mm (range: 60–140), compared to 80 mm (range: 26–147) in non-fistulous cysts. Fistulous cysts were significantly larger than non-fistulous cysts (*p* = 0.040). Notably, the smallest fistulous cyst measured 60 mm in diameter.

When the localization of cysts in the liver was analyzed at the segment level, in the FC group, there were 2 (8.7%) cysts in segment 2, 1 (4.3%) in segment 3, 1 (4.3%) in segment 4b, 1 (4.3%) in segment 5, 2 (8.7%) in segment 6, 7 (30.4%) in segment 7, and 9 (39.1%) in segment 8. On the other hand, in the NFC group, there were 3 (3.3%) cysts in segment 1, 7 (7.6%) in segment 2, 10 (10.9%) in segment 3, 2 (2.2%) in segment 4a, 5 (5.4%) in segment 4b, 15 (16.3%) in segment 5, 19 (20.7%) in segment 6, 16 (17.4%) in segment 7, and 15 (16.3%) in segment 8.

The distribution of cyst types based on the Gharbi classification and the fistula presence is shown in Table 2. In the FC Group, 16 cysts were Type 1, 6 were Type 3, and 1 was Type 5. While surgical intervention is typically not warranted for Type 5 cysts, the patient, in this instance, underwent surgery due to biliary tract compression exerted by the cyst.

**Table 2:** Type and diameter of the cysts according to the presence of cystobiliary fistula

Type, *n* (%)	Cyst Groups		*p-value*
	FC Group (*n* = 23)	NFC Group (*n* = 92)	
1	16(72.7)	67(72.8)	0.409
2	–	6(6.5)	
3	6(27.3)	19(20.7)	
5	1	–	
Diameter (mm)	100 (60–140)	80 (26–147)	0.040
Location (segment), n (%)			
1	–	3 (3.3)	
2	2 (8.7)	7 (7.6)	
3	1 (4.3)	10 (10.9)	
4a	–	2 (2.2)	
4b	1 (4.3)	5 (5.4)	
5	1 (4.3)	15 (16.3)	
6	2 (8.7)	19 (20.7)	
7	7 (30.4)	16 (17.4)	
8	9 (39.1)	15 (16.3)	

*FC* Fistulous cyst, *NFC* Non-fistulous cyst

In the NFC Group, 67 cysts were Type 1, 19 were Type 3, and 6 were Type 2. Although Types 1 and 3 were shared in fistulous and non-fistulous cysts, there was no statistically significant difference in cyst type distribution between the two groups (*p* = 0.583).

### Complications related to cystobiliary fistulas

Post-interventional complications related to cystobiliary fistulas were observed in both surgical and percutaneous procedures as follows:

- *Surgical Patients*: Two patients developed bilomas at the operation site following surgery. One of these patients underwent a second surgery for biliary fistula repair. The other patient received a percutaneous drainage catheter followed by ERCP and sphincterotomy, leading to the resolution of bile drainage. Two other patients experienced bile leakage through the surgical drain postoperatively, which ceased to leak after ERCP and sphincterotomy.
- *Percutaneous Patients*: Of the 15 patients treated percutaneously and four diagnosed with cystobiliary fistulas during the intervention, two required surgical intervention, while the remaining two patients underwent ERCP with sphincterotomy, which successfully resolved bile drainage.

### Albendazole therapy

Albendazole prophylaxis was administered prior to surgical and percutaneous treatments. Patients were instructed to continue albendazole treatment for a minimum period of one month following surgery and three months following percutaneous treatment. The median duration of albendazole use following the treatments was 232 (30–353) days in the FP group and 120 (30–359) days in the FN group (*p* = 0.116). The median duration of total albendazole use was 180 (90–360) days in the FP group and 180 (30–360) days in the FN group (*p* = 0.510). No statistically significant disparities in the duration of albendazole usage between the two groups were present (Table 1).

### Hospitalization and follow-up

Patients in the FP Group had significantly longer hospital stays (median: 32 (range 15–181) days vs. 7 (range 1–15) days in the FN Group; *p* < 0.00001). Follow-up durations did not differ significantly between the patient groups (median: 25 (4–118) vs. 26 (5–118) months; *p* = 0.465) **(**Table 1**).** Following surgical and percutaneous interventions, patients were subjected to serial ultrasonographic monitoring to assess treatment response and the potential for recurrence. This monitoring occurred monthly for six months, transitioning to three-month intervals until one year and then reverting to six-month intervals contingent upon the patient's clinical status. In one patient who underwent surgical treatment, recurrence was observed 3.5 years later in the same location as the previous cyst, which was treated percutaneously. In another patient, recurrence was observed on three separate occasions in different locations from the previous cysts, with the first recurrence occurring 6 months later. These cysts were treated surgically.

## Discussion

Cystobiliary fistulas are among the most significant complications arising from hepatic hydatid cysts, making their management exceptionally challenging [16–18]. This study, analyzing pediatric patients with hepatic hydatid cysts and cystobiliary fistulas, determined that larger cysts were a significant predictor of fistula formation. However, no statistically significant relationships were found between fistula formation and other demographic or clinical factors, such as patient age, gender, place of residence, existing comorbidities, or the number of hydatid cysts in the patient’s liver.

### Risk factors for cystobiliary fistula formation

- *Demographic Characteristics*: Demographic characteristics, including age, gender, and place of residence, showed no significant associations with fistula development. These results complied with a published pediatric case series on liver hydatid cysts with cystobiliary fistulas [17].
- Clinical Features
  o Comorbidities were not associated with fistula development. The lack of substantial variation in comorbidities suggests that cystobiliary fistula formation is predominantly determined by localized cystic factors rather than systemic patient characteristics.
  o *Cyst Size*: The study found a significant association between cystobiliary fistula formation and cyst size; 86.66% of cysts with cystobiliary fistulas were 75 mm or more prominent, with the smallest cyst size associated with a fistula being 60 mm. This finding aligns with previous research, such as the work by Aydın et al., indicating that cysts more prominent than 75 mm in diameter have an increased risk of biliary communication, reporting a rate of 79%, and Demir et al.’s study reporting that cysts more prominent than 69 mm had a higher risk of developing cystobiliary fistulas [5, 17].
  o *Cyst Type*: Despite the established link between cyst size and fistula formation, other factors assessed in this study, such as cyst type, did not show a statistically significant relationship. Given the fundamental role of ultrasonography in diagnosing intra-abdominal hydatid cysts at both the individual and population levels, its diagnostic application using Gharbi’s classification is standard in our clinical practice [19]. The cystic echinococcosis classification system developed by the World Health Organization's Informal Working Group on Echinococcosis (WHO-IWGE) has been widely adopted. The WHO-IGWE classification system closely resembles the Gharbi classification, owing to Gharbi's contribution to its development [2, 20, 21]. Since this retrospective study covered 2002 to 2016, our cohort’s data involved Gharbi’s classification of cysts.
  o Types 1 and 3 cysts, which represent relatively early and active stages of hydatid cyst development according to the Gharbi classification [7, 22], were common in fistulous and non-fistulous cysts. While a study has suggested that cyst types 2 and 3 and the co-presence of multiple-type cysts were associated with occult cystobiliary fistula, our results did not support this claim [23]. The disparity observed in cyst types may be attributed to the limited sample size of this study.

Overall, this study did not observe significant differences in the demographic data between the two patient groups, nor were there differences in cyst types. These results suggest that while patient-specific factors are important, the intrinsic characteristics of the cysts themselves, particularly size, play a more pivotal role in the development of biliary complications.

### Management of hepatic hydatid cysts with cystobiliary fistulas

Both surgical and minimally invasive procedures, such as percutaneous treatments, are widely employed in managing hepatic hydatid cysts, each offering distinct advantages and limitations. Surgical intervention allows for direct visualization and definitive cystobiliary fistula repair if present. In this study, intraoperative identification and successful repair of fistulas were achieved in ten patients. However, surgical management carries a higher risk of complications, as evidenced by four patients in this group who developed bile leakage and biloma formation. These findings are consistent with existing literature, which highlights the significant morbidity associated with surgical treatment, particularly in cases involving large or complex cysts [24].

In contrast, percutaneous treatment offers a less invasive alternative but carries the risk of undiagnosed biliary communication. Among the four patients in this study who underwent percutaneous procedures, two subsequently developed cystobiliary fistulas requiring surgical intervention. These results emphasize the importance of comprehensive preprocedural imaging, including cystography, to identify and mitigate potential biliary complications before percutaneous treatment. Turkyilmaz et al. even advised leaving a catheter within the cyst for two days if the aspirate in preprocedural imaging was clear, waiting for a cystobiliary fistula to reveal itself if present [16].

While percutaneous interventions are generally safe and effective, surgery may be more appropriate for patients with significant biliary communication or large cysts to ensure optimal clinical outcomes. The selection between surgical and percutaneous approaches for managing hepatic hydatid cysts complicated by cystobiliary fistulas should be tailored to individual patient characteristics and cyst-related factors. Recent studies have explored laparoscopic techniques as an intermediate approach, offering the benefits of minimally invasive surgery while maintaining the ability to manage biliary complications effectively [24, 25]. Interestingly, Kanojia reported a laparoscopic technique, described as directly entering the decompressed cyst with the port and safely extracting the hydatid membranes [26]. The technique was used in six patients with no early and late complications; however, it has not been popularized.

Effective hydatid cyst management, particularly those with cystobiliary fistulas, necessitates a multimodal approach integrating diagnostic preoperative/postoperative imaging (ultrasonography, MRI, ERCP) and surgical, percutaneous, and endoscopic interventions [27]. In this study, fistulas identified intraoperatively were successfully repaired surgically, while ERCP with sphincterotomy effectively managed postoperative bile leakage and other fistula-related complications. Notably, two patients in the surgical group and two in the percutaneous group developed postoperative bilomas, requiring further intervention.

The findings of this study align with previous research, which has demonstrated that biliary complications significantly contribute to increased morbidity in hepatic hydatid disease [11]. Our results support the growing evidence advocating endoscopic retrograde cholangiopancreatography (ERCP) in postoperatively diagnosing and managing biliary complications associated with hepatic hydatid cysts [28]. Performing a sphincterotomy with ERCP and placing a stent in the choledochal duct results in the disablement of the sphincter of Oddi, reduction of biliary tract pressure, and the acceleration of the closure of the fistula by allowing bile to flow easily into the duodenum. In the context of biliary fistulas identified after surgical or percutaneous interventions, endoscopic retrograde cholangiopancreatography (ERCP) has been documented to demonstrate enhanced efficacy during the early phase. Endoscopic techniques, particularly ERCP with sphincterotomy, have proven effective in managing postoperative bile leaks. In this study, ERCP facilitated the resolution of bile drainage in several patients, demonstrating its utility as a minimally invasive alternative that can reduce the need for additional surgical procedures. These findings align with existing literature supporting ERCP as a first-line approach for diagnosing and treating biliary complications related to hepatic hydatid cysts [28]. However, surgical intervention remains necessary in large or complex fistulas that fail to respond to endoscopic management.

### Clinical outcomes in cystobiliary fistulas

Patients with cystobiliary fistulas experienced significantly prolonged hospital stays, reflecting the complexity of treatment and the need for extended postoperative care. The extended hospitalization in these cases was primarily attributed to the need for additional interventions, such as surgical fistula repair or ERCP with sphincterotomy, as well as the necessity for prolonged postoperative monitoring to manage potential complications effectively. This observation corroborates prior research indicating elevated morbidity related to biliary complications in hepatic hydatid disease. Therefore, meticulous postoperative surveillance and vigilant assessment for biliary complications are crucial, particularly in patients with significant cystic involvement or those undergoing minimally invasive procedures, as supported by existing research [11].

Postoperative complications, such as biloma formation and persistent bile drainage, are commonly associated with cystobiliary fistulas. Depending on the severity and extent of bile leakage, treatment options for biloma include surgical repair or percutaneous drainage. Identifying the most appropriate treatment approach is crucial for reducing biliary tract pressure and facilitating fistula closure.

### Implications for clinical practice

The findings of this study highlight the critical role of comprehensive preoperative assessment, particularly in evaluating cyst size and anticipating and mitigating the risk of cystobiliary fistulas and other potential complications. Identifying these risks before initiating treatment is essential for optimizing patient outcomes in hepatic hydatid cyst management.

Advanced imaging modalities, such as magnetic resonance cholangiopancreatography (MRCP), offer detailed visualization of the biliary anatomy and potential cystobiliary communications, thereby enhancing surgical planning. Additionally, intraoperative strategies—including the use of protoscolicidal agents and meticulous exploration for occult fistulas—are crucial in reducing postoperative complications.

A multidisciplinary approach, integrating the expertise of surgeons, radiologists, infectious disease specialists, and gastroenterologists, is essential for improving patient care. Collaborative decision-making and individualized treatment strategies can enhance the effectiveness of interventions, minimize complications, and contribute to better overall clinical outcomes in patients with hepatic hydatid cysts complicated by cystobiliary fistulas.

### Limitations

This study has several limitations that should be acknowledged. First, its retrospective design and single-center setting may limit the generalizability of the findings to broader populations. Additionally, the relatively small sample size and the exclusion of conservatively managed patients introduce the potential for selection bias, which may impact the interpretation of results.

## Conclusions

The findings of this study hold significant implications for managing hepatic hydatid cysts, particularly in identifying and addressing cystobiliary fistulas. Preoperative imaging plays a crucial role in evaluating cyst size and its relationship with the biliary system, enabling the anticipation of potential fistula formation.

Postoperative monitoring is imperative, especially in patients with large cysts or complex fistulas, to detect and manage complications such as bile leakage and biloma formation. A multidisciplinary approach is essential for optimizing treatment strategies. Careful selection of the appropriate therapeutic modality, including endoscopic interventions, can minimize the need for repeat surgeries and prolonged hospital stays.

These results underscore the importance of preoperative risk assessment in predicting potential complications associated with hepatic hydatid cysts. No single treatment approach is universally optimal. Instead, personalized treatment plans, guided by a thorough evaluation of individual risk factors, are essential to enhance patient safety and outcomes. Moreover, ensuring informed consent and educating patients about potential risks are fundamental to shared decision-making in clinical practice.

Cystobiliary fistulas constitute a substantial complication, predominantly linked to greater cyst dimensions, mandating prompt identification and individualized treatment approaches. Combining advanced imaging with minimally invasive techniques offers improved diagnosis and treatment of this condition. More extensive multicenter studies are needed to confirm these findings and improve our understanding of risk factors and optimal management of cystobiliary fistulas in children with hepatic hydatid disease. Expanding research in this area will contribute to more comprehensive and evidence-based treatment approaches, ultimately improving patient outcomes.

## Data Availability

No datasets were generated or analysed during the current study.
